# Infiltrating T lymphocytes reduce myeloid phagocytosis activity in synucleinopathy model

**DOI:** 10.1186/s12974-016-0632-5

**Published:** 2016-06-30

**Authors:** Annika Sommer, Tanja Fadler, Eva Dorfmeister, Anna-Carin Hoffmann, Wei Xiang, Beate Winner, Iryna Prots

**Affiliations:** IZKF Junior Research Group 3 and BMBF Research Group Neuroscience, Interdisciplinary Center for Clinical Research, Friedrich-Alexander-Universität (FAU) Erlangen-Nürnberg, Glückstrasse 6, 91054 Erlangen, Germany; Bavarian Research Network on Induced Pluripotent Stem Cells (“ForIPS”), Erlangen, Germany; Institute of Biochemistry (Emil-Fischer-Center), FAU Erlangen-Nürnberg, Fahrstrasse 17, 91054 Erlangen, Germany; Institute of Human Genetics, FAU Erlangen-Nürnberg, Schwabachanlage 10, 91054 Erlangen, Germany

**Keywords:** Parkinson’s disease, Adaptive immune system, mThy1 WTS model, Rag2

## Abstract

**Background:**

Synucleinopathies comprise a group of neurodegenerative diseases associated with abnormal accumulation of α-synuclein. One of the key factors that contribute to the progression of synucleinopathies is neuroinflammation. However, the role of lymphocytes in synucleinopathies like Parkinson’s disease (PD) remains largely unclear.

**Methods:**

To investigate how lymphocytes impact synucleinopathies, human wild-type α-synuclein (WTS) transgenic mice were crossed with mice lacking mature lymphocytes (Rag2^−/−^). In this in vivo model, we quantified α-synuclein aggregation in the substantia nigra (SN) and striatum and determined the numbers of innate and adaptive immune cells in the central nervous system (CNS). The activation state of resident and infiltrated CNS myeloid cells (M1 vs. M2) was further classified by gene and protein expression analyses. The impact of T and B lymphocytes on the phagocytic activity of microglia in the presence of α-synuclein aggregates was addressed in BV2 microglia in vitro.

**Results:**

Compared to WTS^+^ Rag2^+/+^ mice, where T but not B lymphocytes infiltrated the CNS, decreased amounts of α-synuclein aggregates were found in WTS^+^ Rag2^−/−^ mice devoid of mature lymphocytes. The presence of T lymphocytes did not alter the number of Iba1^+^ microglia but increased the frequency of the CD11b^+^ CD45^hi^ population in the CNS, indicative of an increased number of infiltrated macrophages. Moreover, the M1 phenotype was more prominent in WTS^+^ Rag2^+/+^ mice, whereas the M2 activation state was dominating in the absence of lymphocytes in WTS^+^ Rag2^−/−^ mice. In vitro, in the presence of T but not B lymphocytes, significantly less α-synuclein was phagocytosed by BV2 microglia, further supporting the prevalence of the M1 phenotype in the presence of T lymphocytes.

**Conclusions:**

Peripheral T lymphocytes strongly contribute to increased α-synuclein pathology via modulation of CNS myeloid cell function. In the presence of T lymphocytes, microglia phagocytosis of aggregated α-synuclein is reduced, which increases the severity of synucleinopathy.

**Electronic supplementary material:**

The online version of this article (doi:10.1186/s12974-016-0632-5) contains supplementary material, which is available to authorized users.

## Background

Synucleinopathies comprise a group of neurodegenerative diseases characterized by abnormal deposition of α-synuclein in neurons and glia. The most frequent synucleinopathy is Parkinson’s disease (PD), in which α-synuclein pathology propagates throughout the brain as clinical symptoms progress [1]. Possible pathological mechanisms resulting in α-synuclein aggregation and neurodegeneration in sporadic PD are environmental factors, mitochondrial dysfunction, oxidative stress, and neuroinflammation [2, 3]; however, the exact mechanism of α-synuclein aggregation remains elusive.

Myeloid cells collectively describe cells derived from the bone marrow, including granulocytes and monocytes [4]. In the central nervous system (CNS), several myeloid populations are present including microglia and macrophages (in further termed CNS myeloid cells) [5]. Upon activation, in response to brain injuries or to immunological stimuli [6, 7], CNS myeloid cells undergo morphologic alterations from resting ramified CNS myeloid cells into activated amoeboid CNS myeloid cells. Activated CNS myeloid cells are further divided into classical activation (M1 phenotype), characterized by expression of pro-inflammatory genes (e.g., TNF-α, IL-1β, and ICAM), or alternative activation (M2 phenotype), indicating an anti-inflammatory phagocytic phenotype, expressing characteristic phagocytic and anti-inflammatory genes (e.g., Arg1, Lgals3, and CD200r), analogous to peripheral myeloid cells. The M2 phenotype is involved in debris clearance [8–11] and was shown to decrease pathology in multiple sclerosis (MS) and amyloid beta deposits in Alzheimer’s disease [12]. In PD, activated microglia and pro-inflammatory cytokine production were evident in human *post mortem* brains and animal models [13–16], although the modulation of myeloid cell activation in PD is not yet fully understood.

Besides activation of myeloid cells [17], there are indications that the adaptive immune response is also involved in PD-associated disease progression [18, 19]. A genome-wide association study (GWAS) linked sporadic PD with polymorphisms in the human leukocyte antigen (HLA) region, a locus of genes encoding for surface proteins, expressed by activated antigen presenting cells, including microglia in the brain, and interacting with T cell receptors [20]. Alterations in lymphocyte populations were determined in the peripheral blood of PD patients [17, 21]. Furthermore, T lymphocytes were shown to infiltrate the brain of PD patients and to mediate dopaminergic (DA) neuronal loss in the 1-methyl-4-phenyl-1,2,3,6-tetrahydropyridine (MPTP) mouse model of PD [18]. The MPTP model is characterized by acute DA neuronal loss. Besides neuronal loss, continuous aggregation of α-synuclein is the major hallmark of PD pathology, preceding neuronal loss. Therefore, transgenic animal models over-expressing α-synuclein will specifically allow deciphering, whether and how adaptive immune cells are involved in the early pathological mechanism of disease progression in synucleinopathies.

Accordingly, we asked, what is the impact of lymphocytes in a mouse model for synucleinopathies over-expressing human wild-type α-synuclein (WTS) under the murine Thy1 (mThy1) promoter [22]. Therefore, we crossed mThy1 WTS mice (WTS^+^) with mice containing a deletion of the Rag2 gene (Rag2^−/−^), which lack mature lymphocytes [23]. We demonstrate that infiltration of T lymphocytes into the CNS of WTS^+^ Rag2^+/+^ mice increased α-synuclein pathology in the substantia nigra (SN) and striatum, while no B cells were found. The presence of T cells in WTS^+^ Rag2^+/+^ mice was strongly associated with increased levels of pro-inflammatory mediators and the M1 phenotype. In the absence of T cells, increased expression of M2 defining markers and higher frequencies of infiltrating macrophages (CD11b^+^ CD45^hi^) were found in the CNS, which could contribute to the decreased levels of α-synuclein aggregates in WTS^+^ Rag2^−/−^ mice due to increased phagocytic activity. Conversely, B cells did not affect phagocytosis activity of myeloid cells in vitro.

Our data indicate that T lymphocytes aggravate the aggregation of α-synuclein through the modulation of the CNS myeloid cell activation state. This finding will increase the understanding of T cell-mediated inflammation in synucleinopathies.

## Methods

### Animals

Animal experiments were approved by the Bavarian authorities for animal experimentation (TS-2/14). All experiments were performed following the European (2010/63/EU) and National Institute of Health (NIH) Guidelines for the Humane Treatment of Animals. Transgenic mice (tg) over-expressing human WTS under the mThy1 promoter (line 61; kindly provided by Eliezer Masliah, USCD [22]) were crossed with mice carrying a germline mutation, in which a large portion of the coding region of the Rag2 gene is deleted, resulting in a lack of mature B and T lymphocytes (termed Rag2^−/−^; [23]; kindly provided by Jürgen Wittmann, FAU Erlangen-Nürnberg). After several back crossings, WTS^+^ Rag2^+/+^ mice were compared to the WTS^+^ Rag2^−/−^ littermate controls. WTS^−^ Rag2^+/+^ and WTS^−^ Rag2^−/−^ mice were used as additional controls for the effect of α-synuclein over-expression where appropriate. Polymerase chain reaction (PCR) was used to determine the WTS and Rag2 genotype. Animals were kept in a 12-hour (h) light/dark cycle and had access to food and water. For dissection, 22–26-week-old animals were deeply anesthetized by CO_2_ inhalation and transcardially perfused with ice-cold PBS. Brains were removed and either rapidly stored at −80 °C for biochemical and RNA analyses or post-fixed in 4 % PFA for 6 h on ice and subsequently stored in 30 % sucrose in PBS at 4 °C until further processing.

### Immunohistochemistry

Frozen fixed brain hemispheres were cut into 40 μm thick coronal sections using a sliding microtome (Leica SM 2010R) and stored in cryo-protectant solution (25 % ethylene glycol, 25 % glycerol in 0.1 M phosphate buffer, pH 7.4) at −20 °C. Immunohistochemical staining of free-floating sections was performed as published previously [24]. Briefly, sections were washed three times in tris-buffered saline (TBS; pH 7.4) containing 0.05 % Triton X100 prior to a 30-minute (min) treatment with citrate buffer (target retrieval solution, DAKO) at 80 °C. After extensive washing, the sections were blocked for 1–2 h in blocking solution (TBS, 3 % Donkey Serum, 0.03 % Triton X100). Subsequently, sections were incubated with primary antibody overnight at 4 °C using the following primary antibodies: anti-human-α-synuclein (15G7, Enzo, 1:100), anti-CD3 (MCA1477, Serotec, 1:200), or anti-CD19 (115501, BioLegend, 1:200), followed by incubation with secondary antibodies according to the primary host (anti-rat-horseradish peroxidase (HRP), Dianova) for 1 h at room temperature (RT). Amplification of signal intensity was achieved using avidin-biotin peroxidase complex for 1 h (ABC Kit, Vector Laboratories), and signal visualization was reached using 3,3-diaminobenzidine (DAB) as the peroxidase substrate. All sections were mounted in anatomical order on glass slides and coverslipped with NeoMount (Merck). For each staining, the sections of all mice from one experiment were processed simultaneously under the same conditions.

### Immunofluorescence

After blocking as described above, the following primary antibodies were applied to the free-floating sections for a 48 h incubation at 4 °C: anti-tyrosine hydroxylase (TH) (MAB318, Millipore, 1:250), anti-ionized calcium-binding adapter molecule (Iba) 1 (019-19741, WAKO, 1:500), and anti-human-α-synuclein (15G7, Enzo, 1:100). Subsequently, secondary fluorochrome-coupled antibodies (anti-mouse AlexaFluor 488, anti-rabbit Alexa Fluor 567, anti-rat Alexa Fluor 488, all from Life Technologies, 1:500) were added to the sections for 1 h at RT followed by 1 min staining with 4′,6-diamidin-2-phenylindol (DAPI) (10 μg/ml, Life Technologies) to visualize cell nuclei. All the sections were mounted in anatomical order on glass slides and coverslipped with Aqua-Poly/Mount (Polysciences). For each staining, the sections of all mice from one experiment were processed simultaneously under the same conditions.

### Counting procedures

Quantification was achieved using a semiautomatic stereology workstation microscope AxioImager M2 (Carl Zeiss) with a Stereo Investigator 11 software (MicroBrightField) as previously described [25]. All counting procedures were performed on 40-μm thick coronal sections, and every sixth section was analyzed for each experiment. Two different brain regions were analyzed: the SN at the coordinates anterior-posterior (ap) −2.58 to −3.78 mm and the striatum ap 1.42 to −2.28 mm according to the Allen Mouse Brain Atlas (http://mouse.brain-map.org/static/atlas). The respective area was measured using the Stereo Investigator 11 software. All cells within a given area were counted exhaustively in both regions using bright field (for DAB immunostainings of α-synuclein and CD3) and fluorescence (for TH and Iba1 stainings) microscopy. The investigator performing the quantification was blinded to the genotype groups during the analysis.

### Morphological characterization of CNS myeloid cells

The morphological characterization of CNS myeloid cells was evaluated by defining four morphological types based on the morphology of Iba1^+^ CNS myeloid cells. These morphological phenotypes are associated with different states of CNS myeloid cell activation: resting, primed, reactive, and activated [26–29]. Resting CNS myeloid cells (type 1) displayed a small but defined cell body and extensive numbers of branching processes. Type 2 CNS myeloid cells, defined as primed CNS myeloid cells, remained highly ramified but represented a distinctive ellipsoid-like soma. Both type 3 (reactive) and type 4 (activated) CNS myeloid cells presented amoeboid-shaped cell bodies, while the processes were less extensive and shorter. While type 3 CNS myeloid cells displayed few branches, which were generally longer than the cell body diameter, type 4 CNS myeloid cells had no or few unbranched processes seen to be within the length of the cell body diameter [28, 29]. Three pictures visualizing Iba1^+^ cells in the SN region of five mice/group were quantified according to the defined types. At least 30 CNS myeloid cells per mouse were traced, reconstructed, and characterized.

### Flow cytometry

Brains from 22- to 26-week-old animals were homogenized using a 5-ml Glass Tissue Grinder (A. Hartenstein). The homogenized solution was mixed with 100 % Percoll solution (GE healthcare) to a final 70 % Percoll solution and layered under a 30 % Percoll solution. After 30 min centrifugation at 500*g* without brake at RT, the cells were isolated from the 30–70 % layer and washed with PBS. Per staining, 100 000 cells were used. The staining was performed in FACS-PBS (PBS, 2 % FCS, 5 mM EDTA) in the presence of the FcBlocker (Miltenyi Biotec) using the following fluorescently labeled antibodies: anti-CD4-PE, anti-CD8-APC, anti-CD11b-FITC, and anti-CD45-APC (all from Miltenyi Biotec), for 15 min at RT in the dark. Samples were analyzed using a Gallios flow cytometer (Beckman Coulter). Thirty thousand events were recorded. Flow cytometry data were analyzed using the FlowJo 8.5.3 software (FlowJo, LLC). For viability testing, the Fixable Viability Dye eFluor^®^ 780 (eBioscience) was used according to the manufacturer’s instructions. The gating of the living cell population was performed based on the live staining; dead cell populations were excluded. The total cell number was determined utilizing bead measurement (Beckman Coulter CC Size Standard L10), and the absolute cell numbers were calculated according to the manufacturer’s instructions.

### Real-time PCR

Frozen brain tissues were homogenized using an automated TissueLyser LT (Qiagen) according to the manufacturer’s instructions. Total RNA was extracted from homogenized brain hemispheres using the RNeasy Mini kit (Qiagen) with a DNA digestion step (RNase Free DNase Set; Qiagen) according to the manufacturer’s instructions. Five hundred nanograms of total RNA was reversely transcribed into complementary DNA (cDNA) in 20 μl reaction solution using the QuantiTect Reverse Transcription Kit (Qiagen) according to the manufacturer’s instructions. One microliter of cDNA was used per real-time PCR (RT-PCR) reaction. RT-PCR was performed in duplicates in a final volume of 20 μl using either 1× TaqMan Universal Master Mix II, no UNG and 1× TaqMan Gene Expression Assay for GAPDH, or 1× SYBR Green PCR Master Mix (all from Applied Biosystems) and 200 nM of each primer for all other transcripts in the ABI PRISM 7300 Sequence Detection System (Applied Biosystems). Forward and reverse primer pairs (Sigma-Aldrich) for SYBR Green PCR are shown in Table 1.

**Table 1:** Primer sequences for RT-PCR analysis

Gene	Forward (5′–3′)	Reverse (5′–3′)
TNF-α	TCTTCTCATTCCTGCTTGTGG	GGTCTGGGCCATAGAACTGA
Hmox1	GTCAAGCACAGGGTGACAGA	ATCCCTGCAGCTCCTCAAA
ICAM1	GTGATGCTCAGGTATCCATCCA	CACAGTTCTCAAAGCACAGCG
IL-1β	CTGTGACTCATGGGATGATGATG	CGGAGCCTGTAGTGCAGTTG
Arg1	GAATCTGCATGGGCAACC	GAATCTGGTACATCTGGGAAC
Cxcr1	AAGTTCCCTTCCCATCTGCT	CAAAATTCTCTAGATCCAGTTCAGG
Lgals3	AAGGAGAACAGGGAAAGG	TGGACTTGCAGGGCTTCT
CD200r	AAATGCAAATTGCCAAAATTAGA	GTATAGTAGCATAAGGCTGCATTT
Trem2	TGGGACCTCTCCACCAGTT	GTGGTGGTGTTGAGGGCTTGG
HPRT1	TCAGTCAACGGGGACATAAA	GGGGCTGTACTGCTTAACCAG

The RT-PCR program was as follows: 95 °C for 10 min, followed by 40 cycles at 95 °C for 15 s and 60 °C for 1 min and 1 cycle at 95 °C for 15 s, 60 °C for 30 s and 95 °C for 15 s. The specificity of the SYBR Green detection was controlled by the occurrence of a single peak in the dissociation curve. Relative quantification was performed by calculating the difference in cross-threshold values (ΔCt) of the gene of interest and a mean of two housekeeping genes, GAPDH and HPRT1, according to the formula 2^−ΔCt^.

### Western blot analysis

Protein levels were assessed in brain lysates by western blot analysis. Briefly, homogenized brains were lysed in lysis buffer (2 % NP40, 150 mM NaCl, 50 mM HEPES, 10 % Glycerol, pH 8.5) and a cocktail of protease inhibitors (complete Mini EDTA free, Roche). Protein concentrations of lysed samples were determined using the bicinchoninic acid assay (Pierce BCA Protein Assay Kit, Thermo Fisher Scientific). Equal amounts of total protein (60 μg) were separated on 10 % SDS PAGE and transferred to nitrocellulose membranes (A. Hartenstein). Blots were probed using primary antibodies: anti-CD206 (R&D Systems, 1:2000), anti-ß-actin (Sigma-Aldrich, 1:3000), anti-α-synuclein (BD Transduction Laboratories 1:2000), and anti-GAPDH (FL-335, Santa Cruz Biotechnology, 1:1000), and HRP-conjugated anti-mouse, anti-rabbit, and anti-goat secondary antibodies (Dianova, 1:20,000). For visualization, the ECL SuperSignal West Pico or Femto (Thermo Scientific) and ChemiDoc™ XRS+ System (BioRad) were used. Signal intensity was measured by the ImageLab software (version 5.2.1). The intensity of CD206 was normalized to that of actin.

### BV2 co-culture

BV2 microglia (kindly provided by Johannes Schlachetzki, FAU Erlangen-Nürnberg) were cultured in DMEM/F12 + Glutamax medium supplemented with 100 U/ml penicillin/streptomycin (both from Thermo Fisher Scientific) and 10 % heat-inactivated FCS (1 h 56 °C). α-synuclein aggregates were generated by incubating recombinant human α-synuclein (100 μM in PBS; kindly provided by Dr. Silvia Campioni, Swiss Federal Institute of Technology Zurich, Switzerland) for 6 h at 37 °C with slight agitation at 500 rpm, followed by 12 h shaking at 56 °C, 500 rpm. Primary T and B lymphocytes were isolated from spleens of WTS^+^ Rag2^+/+^ and WTS^−^ Rag2^+/+^ mice. Briefly, isolated spleens were homogenized in PBS/2 % FCS using a 70-μm strainer. After centrifugation of the homogenate (5 min at 500 g), the pellet was incubated in erythrocyte lysis buffer (0.15 M NH_4_Cl, 20 mM HEPES) for 5 min at RT. Subsequently, the reaction was stopped with PBS/2 % FCS, followed by centrifugation for 5 min at 500 g. The resulting pellet was used for CD3 and CD19 MACS sorting (CD3ε MicroBead Kit, mouse; CD19 MicroBead Kit, mouse; both Miltenyi Biotec) according to the manufacturer’s instructions. Isolated CD3^+^ T lymphocytes were activated (Dynabeads^®^ Mouse T-Activator CD3/CD28; Thermo Fischer Scientific) and directly used for co-culture with BV2 cells. B lymphocytes were activated with lipopolysaccharide (LPS, 10 μg/ml, Sigma-Aldrich) for 2 h prior co-culture with BV2 cells. All co-cultures were performed in a ratio of 1:10 (lymphocytes/BV2 cells) in the presence of 200 nM aggregated α-synuclein. After 24 h, the co-cultured cells were fixed and analyzed for Iba1^+^ and α-synuclein^+^ by immunofluorescence staining (Iba1, WAKO, 1:300; α-synuclein 15G7, Enzo, 1:200). For evaluation, the LSM 780 confocal laser scanning microscope (×63 PLAPOoil objective, pinhole 1 airy unit; Carl Zeiss) was employed, and cells were quantified using ImageJ software. The phagocytosis level was determined as the frequency of Iba1 and α-synuclein double-positive cells over the total Iba1^+^ cell amount in the co-culture. Three independent experiments were performed.

### Statistical analysis

Each in vivo experiment was performed with ten mice for stereology experiments and three to five mice/group for flow cytometry, immunofluorescence staining, and gene and protein expression as well as for BV2 co-culture experiments. Differences between the two groups were analyzed using the two-tailed Student’s *t* test. When more than two groups were compared, differences were analyzed with a one-way ANOVA followed by Tukey post hoc test. In all analyses, *p* values of less than 0.05 were considered significant. All statistical tests were conducted with GraphPad Prism 5 software (GraphPad Software, Inc.).

## Results

### The presence of lymphocytes exacerbates α -synuclein aggregates in the SN and striatum

**Fig. 1:**
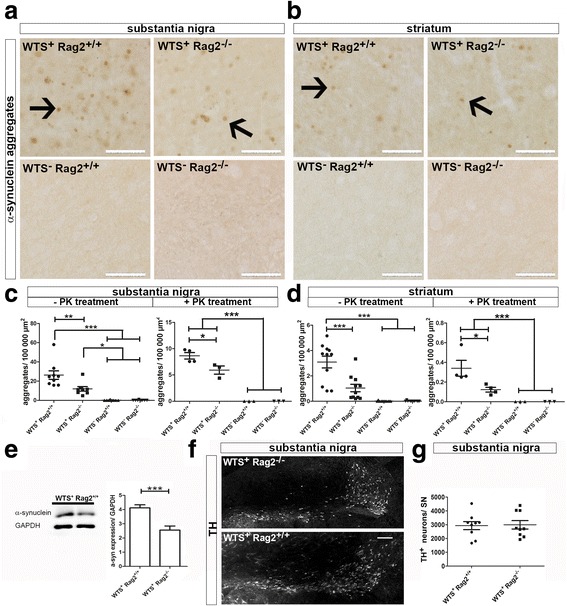
Increased numbers of α-synuclein in the presence of lymphocytes. **a**, **b** Representative bright field pictures of the **a** substantia nigra and **b** striatum show α-synuclein aggregates in WTS^+^ Rag2^+/+^ and WTS^+^ Rag2^−/−^ but not in WTS^−^ mouse brains. α-synuclein was stained in the brain tissue of 22–26-week-old mice using anti-α-synuclein (15G7) antibody. *Scale bars* 100 μm. *Arrows* indicate α-synuclein aggregates determined as brown spots of at least 3 μm in diameter and are shown by arrows. **c**, **d** Significantly reduced numbers of α-synuclein aggregates were found in WTS^+^ Rag2^−/−^ compared to WTS^+^ Rag2^+/+^ mice in both the substantia nigra and striatum without and with proteinase K (PK) treatment. Control mice (WTS^−^) did not show any aggregates. T-test **p < 0.01, ***p < 0.001. **e** The protein level of α-synuclein was determined by western blotting indicating increased amount of α-synuclein protein in WTS^+^ Rag2^+/+^ mice compared to WTS^+^ Rag2^−/−^ mice (*n* = 3 per group). **f** WTS^+^ Rag2^+/+^ and WTS^+^ Rag2^−/−^ brains were stained for the dopaminergic neuronal marker tyrosine hydroxylase (TH), and representative pictures of the substantia nigra are shown. **g** No significant difference in the number of TH^+^ neurons could be detected by stereological quantification in the SN of WTS^+^ Rag2^+/+^ compared to WTS^+^ Rag2^−/−^ mice. *Scale bar* 50 μm. **c**, **d**, **g** Data from nine to ten (five for WTS^−^) mice/group are shown

We addressed the role of adaptive immune cells in synucleinopathies. Therefore, mice over-expressing human WTS (WTS^+^) [22] were crossed with mice lacking mature T and B lymphocytes (Rag2^−/−^) [23]. Brain tissues of WTS^+^ Rag2^+/+^ and WTS^+^ Rag2^−/−^ mice (*n* = 10) were stained for α-synuclein. α-synuclein aggregates, defined as distinct brown spots of at least 3 μm in diameter (Fig. 1a, b, arrows), were counted stereologically in the PD-associated regions SN and striatum. In WTS^+^ Rag2^+/+^ and WTS^+^ Rag2^−/−^ mice, α-synuclein aggregates were frequently detected both in the SN and striatum (Fig. 1a, b). Interestingly, in mice with intact lymphocytes (WTS^+^ Rag2^+/+^), significantly more α-synuclein aggregates were determined (26 ± 4 aggregates/100,000 μm^2^ SN) as compared to WTS^+^ Rag2^−/−^ mice lacking lymphocytes (12 ± 5 aggregates/100,000 μm^2^ SN) (Fig. 1c, d). In line, proteinase K (PK) treatment indicated significantly increased amounts of insoluble PK-resistant α-synuclein aggregates in the SN and striatum in the presence of mature lymphocytes in WTS^+^ Rag2^+/+^ mice (8 ± 1 aggregates/100,000 μm^2^ SN) compared to WTS^+^ Rag2^−/−^ mice (6 ± 1 aggregates/100,000 μm^2^ SN) (Fig. 1c, d). Control mice (WTS^−^ Rag2^+/+^ and WTS^−^ Rag2^−/−^ (*n* = 3 per group)) did not show any α-synuclein aggregates (Fig. 1a–d). Since α-synuclein pathology in the SN was found to be more prominent than in the striatum, further analyses focused on the SN. Confirming the immunohistochemical analysis, western blot analysis also showed lower amounts of α-synuclein protein in absence of lymphocytes (Fig. 1e).

To analyze, whether lymphocytes affect the number of dopaminergic neurons, we stained for TH^+^ neurons in the SN (Fig. 1e). No differences in the number of TH^+^ neurons in WTS^+^ Rag2^+/+^ mice (2 927 ± 290 TH^+^ neurons/SN) compared to WTS^+^ Rag2^−/−^ mice (2 989 ± 306 TH^+^ neurons/SN) were observed (Fig. 1f). In accordance with previous publications [22, 30], there was no significant reduction of TH^+^ neurons in the SN between WTS tg (WTS^+^ Rag2^+/+^, WTS^+^ Rag2^−/−^) and non-tg (WTS^−^ Rag2^+/+^, WTS^−^ Rag2^−/−^) mice (data not shown). Together, our data indicate that lymphocytes exacerbate α-synuclein aggregation in a progressive synucleinopathy model.

### T lymphocytes infiltrate the brain of WTS^+^ Rag2^+/+^ mice

**Fig. 2:**
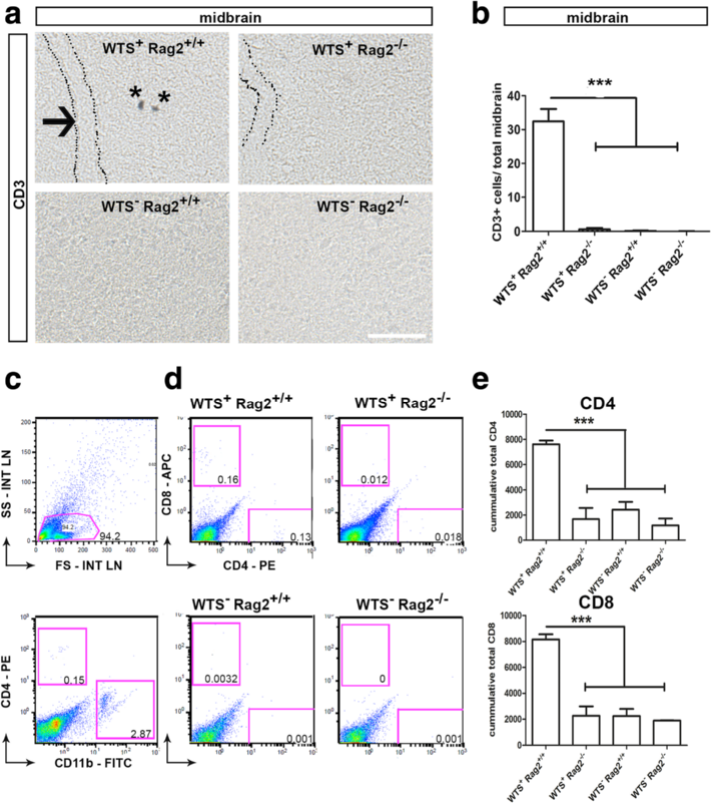
CD3^+^ lymphocytes infiltrate the midbrain of WTS^+^ Rag2^+/+^ mice. **a** WTS^+^ Rag2^+/+^ and WTS^+^ Rag2^−/−^ as well as WTS^−^ brains were stained for the T lymphocyte marker CD3 as shown in representative bright field pictures of the midbrain and the quantification in **b**. Infiltrating CD3^+^ lymphocytes, labeled with *asterisks*, do not co-localize with blood vessels (indicated by the *dotted lines*). CD3^+^ cells were only present in WTS^+^ Rag2^+/+^ mice, but not in WTS^+^ Rag2^−/−^ or WTS^−^ mice. *Scale bar* 50 μm. **b** Data from five mice/group are shown. **c–e** Flow cytometry analysis of the brain tissues was performed to confirm the presence and to analyze the subsets of T lymphocytes in WTS^+^ Rag2^+/+^ mice. **c** 30,000 events were recorded, and a total population was defined in the forward/side scatter, excluding dead cells, which were identified by utilizing a live-dead staining, for further gating strategies (*upper histogram*). The CD4^+^ (CD11b^−^) population representing CD4^+^ T lymphocytes and distinguished from CD4 low positive CD11b^+^ microglia population is shown in a representative dot plot (*lower histogram*). Representative scatter and dot plot are shown from brain tissue of a WTS^+^ Rag2^+/+^ mouse. **d** Gated populations for the T lymphocyte subset markers CD4 (T helper cell) and CD8 (cytotoxic T cell) are visualized in representative dot plots, and total cell numbers of CD4 and CD8 T cells per group ± SEM are shown in **e**, which were determined based on bead measurement and calculation of the total cell number. Significantly more CD4^+^ and CD8^+^ T cells are present in WTS^+^ Rag2^+/+^ compared to WTS^+^ Rag2^−/−^ and WTS^−^ mouse brain tissues. Data from five mice/group are shown. T-test, *p < 0.05, ***p < 0.001

A previous study emphasized a crucial role of T lymphocytes rather than B cells in mediating pathology in the MPTP mouse model of PD [18]. To analyze the presence of T and B lymphocytes in the CNS in WTS^+^ and WTS^−^ mice, we investigated lymphocyte infiltration into the brain tissue by immunohistochemical staining for the T lymphocyte marker CD3 and the B lymphocyte marker CD19. Positively stained CD3 cells were found in low numbers and broadly distributed in the midbrain of WTS^+^ Rag2^+/+^ mice but not in mice of WTS^+^ Rag2^−/−^ nor WTS^−^ mice (Fig. 2a, b). No positive staining for CD19 was detected in the midbrain of WTS^+^ mice (Additional file 1). Further analysis of the infiltrating T lymphocyte population was performed in whole-brain homogenates. Flow cytometry revealed the presence of both CD4^+^ (helper) and CD8^+^ (cytotoxic) T lymphocytes in brains of WTS^+^ Rag2^+/+^, but not in brains of WTS^+^ Rag2^−/−^ or in WTS^−^ mice (Fig. 2c–e).

### Infiltrating T lymphocytes are associated with a pro-inflammatory M1 phenotype

**Fig. 3:**
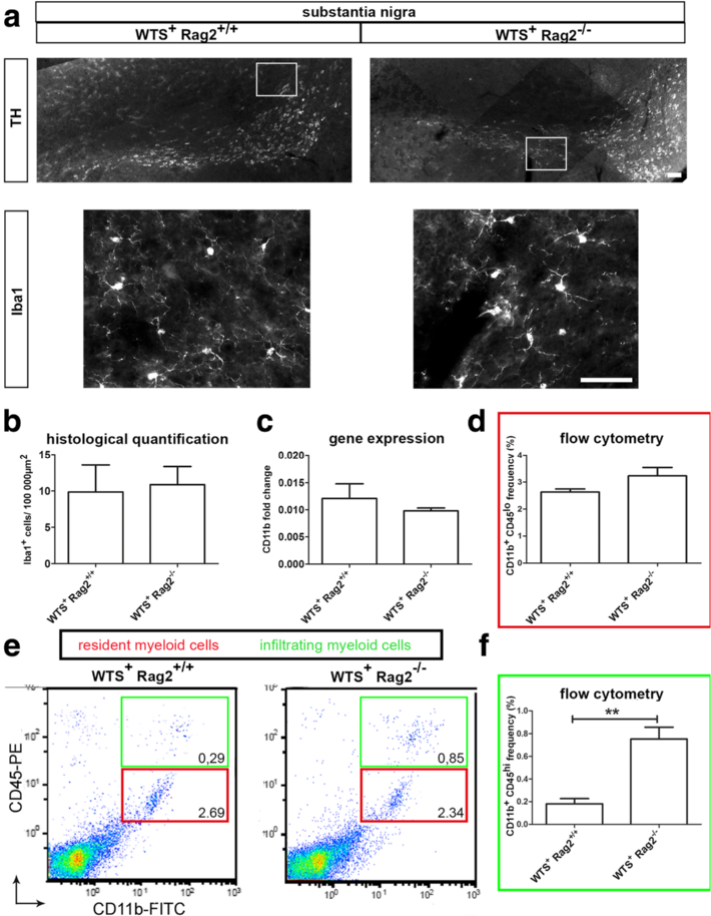
Increased presence of infiltrating myeloid cells in brains of WTS+ Rag2^−/−^ mice. **a** Representative fluorescence pictures of the substantia nigra (SN) of WTS^+^ Rag2^+/+^ and WTS^+^ Rag2^−/−^ mice stained for TH^+^ neurons to visualize the SN (*upper panel*) and for Iba1 to visualize CNS myeloid cells in the SN (*lower panel*). *Scale bars* 50 μm. The total number of **b** Iba1^+^ myeloid cells in the SN, **c** CD11b mRNA expression measured by qRT-PCR and **d** CD11b^+^ CD45^lo^ cell frequency measured by flow cytometry (total of 30,000 recorded events, total brain cell population was defined as shown in Fig. 2c, defined as shown in the representative dot plots in **e**, *red box*) were unchanged between WTS^+^ Rag2^+/+^ and WTS^+^ Rag2^−/−^ mouse brain tissues. **e**, **f** A significant increase in infiltrating myeloid cells defined as CD11b^+^ CD45^hi^ population (green box in E) was found in WTS^+^ Rag2^−/−^ compared to WTS^+^ Rag2^+/+^ brains in flow cytometry analysis. Data from four to five mice/group are shown as means ± SEM. T-test, **p < 0.01

To analyze how infiltrating T lymphocytes aggravate α-synuclein aggregation, we examined if infiltrating T lymphocytes influence the CNS innate immune cells. We investigated the possible effect of T lymphocytes on CNS myeloid cells (microglia and brain macrophages), which are responsible for sensing, clearing, and performing the primary immune responses in the CNS environment. To quantify CNS myeloid cells, we first counted Iba1^+^ cells in the SN and did not find differences in the number of Iba1^+^ cells or gene expression of CD11b between WTS^+^ Rag2^+/+^ and WTS^+^ Rag2^−/−^ mice (Fig. 3a–c). We also did not find any significant differences in the morphology of Iba1^+^ cells between WTS^+^ Rag2^+/+^ and WTS^+^ Rag2^−/−^ mice (see Additional file 2: evaluation of microglia morphologies for resting, primed, reactive, and activated microglia). Flow cytometry determined no difference in the frequencies of CD11b^hi^CD45^lo^ cells as marker for resident myeloid cells (Fig. 3d). Interestingly, we found elevated frequencies of CD11b^+^CD45^hi^ cells representing newly infiltrated myeloid cells [31] in WTS^+^ Rag2^−/−^ mice compared to WTS^+^ Rag2^+/+^ (Fig. 3e, f). These data indicate that a lack of mature T lymphocytes led to increased infiltration of peripheral myeloid cells in synucleinopathies, while the frequencies of resident myeloid cells remained unchanged.

**Fig. 4:**
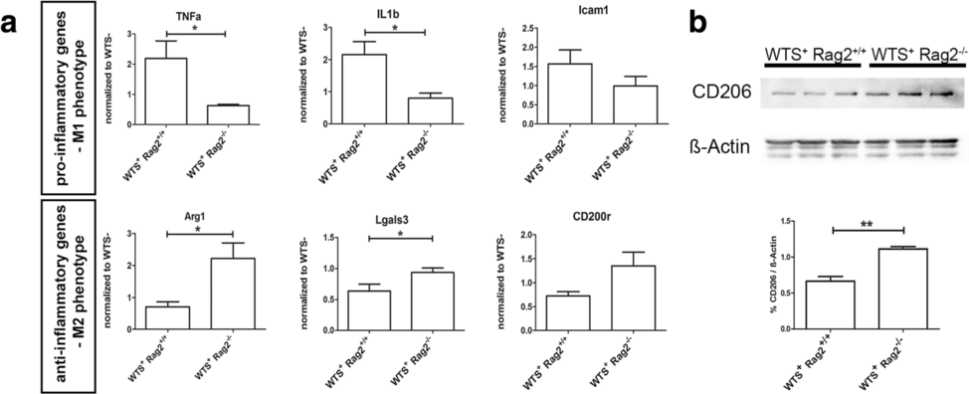
Infiltrating T lymphocytes are associated with a M1 myeloid phenotype. **a** Gene expression of characteristic M1 markers (TNF-α, IL-1β, ICAM1) measured by qRT-PCR shows their upregulation in WTS^+^ Rag2^+/+^ mice compared to WTS^+^ Rag2^−/−^ mice, while genes associated with an anti-inflammatory M2 phenotype (Arg1, Lgals3, CD200r) are upregulated in WTS^+^ Rag2^−/−^ compared to WTS^+^ Rag2^+/+^ mice. The gene expression values in WTS^+^ mice were normalized to the respective gene expression levels in WTS^−^ mice. RNA was extracted from the whole-brain lysates. Data are shown as mean of five mice/group ± SEM. **b** The phagocytic receptor, CD206, is increased in brain tissue of WTS^+^ Rag2^−/−^ compared to WTS^+^ Rag2^+/+^ mice on protein level as shown by western blot analysis with three mice/group (*upper panel*) and by densitometric quantification (*lower panel*) Data are presented as mean ± SEM

Activated CNS myeloid cells are a characteristic feature of synucleinopathies [16, 32]. We further analyzed whether T lymphocytes affect the phenotype of activated CNS myeloid cells. We therefore performed gene expression analysis of characteristic M1 and M2 phenotype markers in the whole-brain lysates of WTS^+^ and WTS^−^ mice and normalized the expression in WTS^+^ Rag2^+/+^ and WTS^+^ Rag2^−/−^ mice to a homeostatic expression of these genes in control WTS^−^ Rag2^+/+^ and WTS^−^ Rag2^−/−^ mice. We found a significant upregulation of M1-associated pro-inflammatory genes including TNF-α and IL-1β and a trend towards more ICAM1 in WTS^+^ Rag2^+/+^ compared to WTS^+^ Rag2^−/−^ mice (Fig. 4a, left panel). In contrast, mice lacking lymphocytes (WTS^+^ Rag2^−/−^) showed significantly upregulated expression levels of the M2-associated anti-inflammatory genes Arg1 and Lgals3 and a tendency for CD200r level compared to WTS^+^ Rag2^+/+^ (Fig. 4a, right panel). Further we analyzed protein expression of CD206 (mannose receptor), which is a phagocytic receptor predominantly associated with M2 phenotype, in brain lysates of WTS^+^ Rag2^+/+^ and WTS^+^ Rag2^−/−^ mice. As shown in Fig. 4b, we could detect significantly increased levels of CD206 in WTS^+^ Rag2^−/−^ brains compared to WTS^+^ Rag2^+/+^ brains, suggesting the prevalence of the M2 phenotype in WTS^+^ Rag2^−/−^ mice. Thus, in the presence of lymphocytes (WTS^+^ Rag2^+/+^), CNS myeloid cells adopt a characteristic M1 phenotype, while in the absence of lymphocytes (WTS^+^ Rag2^−/−^), an M2 phenotype is more predominant.

### BV2 microglia show increased phagocytosis of α-synuclein in the absence of T lymphocytes in vitro

**Fig. 5:**
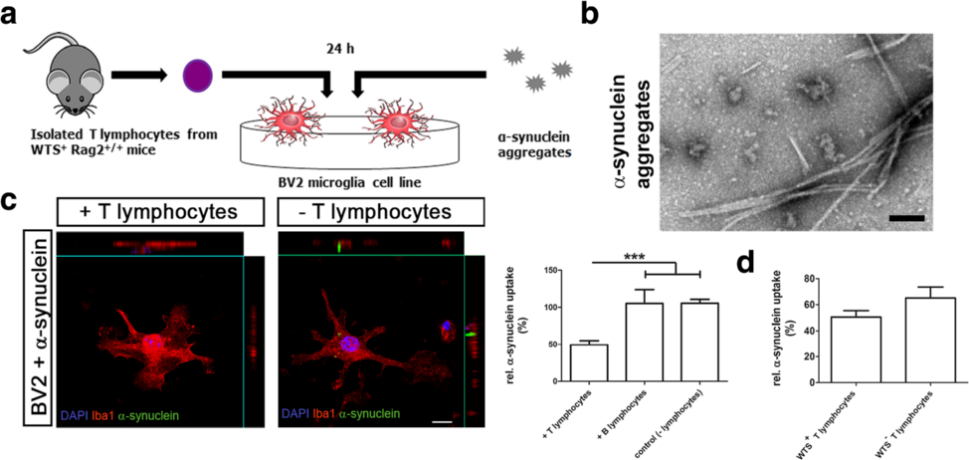
T lymphocytes reduce phagocytosis activity of microglia in vitro. **a** BV2 microglia cells were co-cultivated with aggregated α-synuclein (200nM) in absence or presence of T or B lymphocytes at the 10:1 ratio (BV2/lymphocytes) for 24 h. **b** Recombinant human α-synuclein was aggregated in vitro (electron microscopy picture, *scale bar* 0.2 μm). **c** α-synuclein uptake was quantified as percentage of Iba1 and α-synuclein double-positive cells within total Iba1+ population and normalized to a control uptake level in the absence of lymphocytes. α-synuclein uptake was significantly decreased in the presence of T cells, but not B cells, compared to control condition. **d** Co-culture of BV2 cells under the same conditions as in **c** with T lymphocytes isolated from WTS^−^ mice did not alter the α-synuclein uptake compared to the co-culture with T lymphocytes from WTS^+^ mice. *Scale bar* 20 μm. T-test, *p < 0.05, ***p < 0.001

**Fig. 6:**
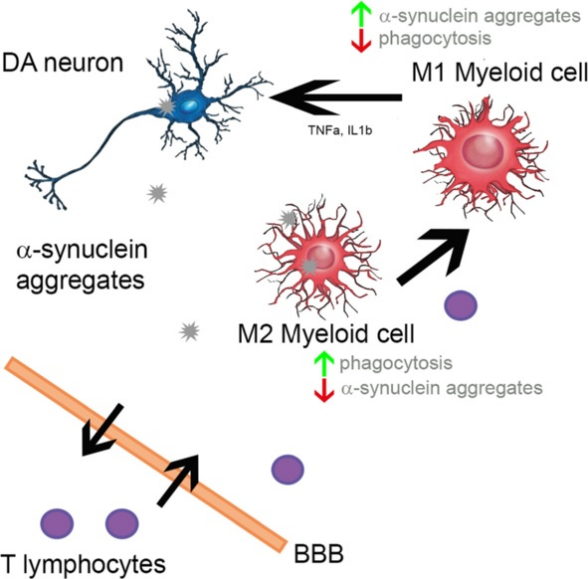
Model of the potential contribution of T lymphocytes in synucleinopathies. In the synucleinopathy, T lymphocytes infiltrate the CNS and lead to an increase of the M1 phenotype of activated myeloid cells. The phagocytic activity of the CNS myeloid cells is thereby reduced leading to the increased presence of α-synuclein aggregates

Since the M2 anti-inflammatory phenotype is linked with higher phagocytic activity compared to the M1 pro-inflammatory phenotype, the effect of T and B lymphocytes on the phagocytic activity of microglia cells was further investigated. We performed an in vitro experiment by co-culturing BV2 microglia cells with α-synuclein aggregates (Fig. 5a) and activated T or B lymphocytes isolated from spleens of WTS^+^ Rag2^+/+^ or WTS^−^ Rag2^+/+^ mice. In the presence of T lymphocytes derived from WTS^+^ Rag2^+/+^ mice, less α-synuclein aggregates were taken up by BV2 cells (Fig. 5c). Similarly, T lymphocytes from WTS^−^ Rag2^+/+^ mice also reduced α-synuclein uptake of BV2 cells (Fig. 5d), demonstrating that the inhibitory effect of T lymphocytes on BV2 phagocytic capacity is rather a specific T cell function. The presence of α -synuclein in T lymphocytes from WTS over-expressing mice did not alter the result. The addition of B lymphocytes did not have any effect on the uptake of α-synuclein aggregates by BV2 cells independently of their origin (either isolated from WTS^+^ Rag2^+/+^ or WTS^−^ Rag2^+/+^ mice) (Fig. 5c). These results show that T lymphocytes, but not B lymphocytes, in the presence of α-synuclein aggregates, reduce the phagocytic activity of microglia. Together, our data suggest a crucial role of T lymphocytes in modulating myeloid cell activation towards a pro-inflammatory M1 phenotype in synucleinopathy (Fig. 6).

## Discussion

While T lymphocytes were recently shown to play an important role for TH^+^ cell loss in a toxin-induced PD mouse model [18, 33], the effect of T lymphocytes for progressive synucleinopathies, and the mechanism of their action remain elusive. In this study, we describe the impact of T lymphocytes in a WTS over-expression synucleinopathy model. The presence of lymphocytes was associated with an increased number of α-synuclein aggregates in the SN and striatum of WTS^+^ Rag2^+/+^ mice and CD3^+^ T lymphocytes. No CD19^+^ B cells were found in the midbrain of these mice. In the absence of lymphocytes, increased amounts of infiltrated myeloid cells (CD11b^+^ CD45^hi^) were detected, which, together with resident microglia, could contribute to the prevalence of the M2 phenotype. Higher expression of the phagocytic receptor CD206 in WTS^+^ Rag2^−/−^ mouse brains, compared to that in WTS^+^ Rag2^+/+^ mice, might indicate increased phagocytosis activity and improved clearance of α-synuclein aggregates in the absence of lymphocytes, supporting the M2 phenotype. Moreover, primary T but not B lymphocytes inhibited the uptake of α-synuclein aggregates by BV2 microglia cells in vitro, indicating a lower phagocytic capacity of CNS myeloid cells in the presence of T cells. Thus, we suggest that T lymphocytes contribute to the progression of synucleinopathies by modulating the myeloid phagocytosis activity (Fig. 6).

### Increased α-synuclein pathology in the SN and striatum in the presence of lymphocytes

α-synuclein aggregation in the mThy1 WTS mouse model is present in various brain regions including the thalamus, basal ganglia, brainstem, SN, and striatum [22]. We analyzed α-synuclein aggregates in the SN and striatum of WTS^+^ Rag2^+/+^ and WTS^+^ Rag2^−/−^ mice. We found that aggregate numbers were significantly decreased in the absence of lymphocytes. Accordingly, a previous study using an acute MPTP mouse model of PD [18] demonstrated a critical contribution of T lymphocytes in dopaminergic cell loss in the SN. Another study showed an early infiltration of CD4 and CD8 T lymphocytes in the AAV-based rat model of PD, over-expressing high levels of human α-synuclein causing neuronal cell death [29]. In accordance with our data, the mThy1 WTS model was previously characterized by the absence of TH^+^ neuronal loss [22, 30] or motor deficits associated with DA neuronal loss [34]. A property of most current PD genetic mouse models is the lack of dopaminergic loss. Loss of TH^+^ neurons is thought to be a late pathological hallmark in PD, while α-synuclein aggregation starts earlier [1]. Thus, using a model of α-synuclein pathology rather than a massive TH^+^ loss may enable investigation of early mechanisms of synucleinopathy.

Taking together, we demonstrated that lymphocytes enhance α-synuclein PD-related pathology as shown by the presence of more α-synuclein aggregates in the SN and striatum. The present study is demonstrating an association between the presence of T lymphocytes and progressively developing α-synuclein pathology.

### CD3^+^ lymphocytes infiltrate the CNS in the synucleinopathy model

A more detailed analysis of the adaptive immune cells in WTS^+^ Rag2^+/+^ mice revealed that CD3^+^ T but not CD19^+^ B lymphocytes infiltrated the midbrain of these mice, suggesting that specifically T lymphocytes influence α-synuclein pathology. Another study using this mThy1 WTS mouse model did not find any increase in the CD8^+^ T lymphocyte infiltration in the striatum [35], possibly due to the brain region specificity of lymphocyte infiltration or due to a preferential CD4 T cell infiltration into the midbrain and striatum. In accordance with our data, Brochard and colleagues could show that CD4^+^ T cells rather than B lymphocytes were critically involved in the progression of disease pathology and were responsible for the elevated neurotoxic effect on DA neurons [18]. Their study also described the presence of T lymphocytes in human *post mortem* brains of PD patients, strongly emphasizing a crucial role of T cells in the pathogenesis of PD. Besides, earlier studies have shown alterations in lymphocyte populations in PD peripheral blood compared to controls [17, 21] and a significant increase of CD4 and CD8 T cells in the blood of mThy1 WTS mice [35]. These findings further stress the importance of peripheral lymphocytes in PD. In addition, in PD models damage of the blood brain barrier was shown [36, 37], allowing the infiltration of peripheral immune cells.

### Increased macrophage infiltration in the CNS of WTS tg mice in the absence of lymphocytes

To investigate the mechanism by which infiltrating CD3^+^ T lymphocytes affect the disease pathology, we examined innate immune cells. Lymphocytes did not influence the quantity of resident myeloid cells as determined by the numbers of Iba1^+^ and frequencies of CD11b^+^CD45^lo^ cells. In addition, we could not detect any differences in the morphology of myeloid cells in the brains of WTS^+^ mice with or without lymphocytes. This suggests that CNS myeloid cells are activated in both WTS^+^ Rag2^+/+^ and WTS^+^ Rag2^−/−^ mice. However, activated CNS myeloid cells can be further distinguished into M1 and M2 currently merely using gene expression analysis of pro- and anti-inflammatory markers. Thus, although we did not observe morphological differences, it might be possible that the activated CNS myeloid cells in our model perform different functions in presence or absence of T lymphocytes. Interestingly, in the AAV-based rat model of PD exhibiting a profound neurodegeneration and cell death, the sustained CNS myeloid cell activation was associated with a prominent T lymphocyte infiltration into the SN [29].

In contrast, increased frequencies of CD11^+^CD45^hi^ cells, a characteristic expression pattern of peripheral myeloid cells [31], were detected in the CNS of WTS^+^ Rag2^−/−^ mice. These findings suggest that there is more intense infiltration of peripheral myeloid cells in WTS^+^ Rag2^−/−^ mice. Infiltration of peripheral myeloid cells was also described for other neurodegenerative diseases like Alzheimer’s disease [38, 39]. Here, we show that this infiltration might be strongly dependent on lymphocytes. Importantly, infiltrating myeloid cells are suggested to primarily eliminate protein aggregates in the brain [40]. In our model, CNS-infiltrated myeloid cells with increased phagocytosis activity [41] could result in a better clearance of α-synuclein aggregates, thereby decreasing pathology in WTS^+^ Rag2^−/−^ mice.

### The M1 phenotype is associated with the presence of T lymphocytes

We could show that, in the presence of T lymphocytes in WTS^+^ Rag2^+/+^ mice, activated CNS myeloid cells had a M1 phenotype, due to increased expression of pro-inflammatory cytokines like TNF-α and IL-1β and reduced protein levels of the phagocytic receptor CD206. In contrast, the lack of lymphocytes in WTS^+^ Rag2^−/−^ mice correlated with increased expression of genes associated with the M2 phenotype and higher expression of CD206, suggesting stronger phagocytic activity in the CNS in these mice. Increased phagocytosis of M2 myeloid cells [8, 9, 32, 42] leading to more efficient clearance might be responsible for the reduced numbers of α-synuclein aggregates found in WTS^+^ Rag2^−/−^ mice. This suggests that M2 myeloid cells are able to reduce α-synuclein pathology and thus could slow down disease progression. On the other hand, if infiltrating T lymphocytes modulate the myeloid cell activation towards a pro-inflammatory M1 phenotype with reduced phagocytic activity, it could explain higher α-synuclein aggregation in WTS^+^ Rag2^+/+^ mice. Consistent with this finding, α-synuclein uptake by BV2 microglia in vitro was reduced in the presence of T lymphocytes independent of their origin, further supporting our results that infiltrating T lymphocytes facilitate the switch from M2 into M1 phenotype in synucleinopathy. On the other hand, α-synuclein uptake by BV2 microglia in vitro was not influenced by the presence or absence of B lymphocytes. Although BV2 cells are frequently used as in vitro cell model to study microglia function [43], a recent study demonstrated the limits of BV2 cells when compared to primary microglia [44]. Therefore, further experiments using primary isolated microglia from mouse brain tissue will be important to study T lymphocyte-dependent α-synuclein uptake in microglia/CNS myeloid cells.

The destructive role of the M1 myeloid cell activity in neurodegenerative diseases was described recently by increased cell death of spinal cord motor neurons in conditions of downregulated M2 population in spinal cord injury [12] and by a more progressive experimental autoimmune encephalomyelitis, the model of MS, under dominating M1 state [45]. Thus, pharmacologically targeting specific myeloid cell activation states might open additional, more effective treatment options for neurodegenerative diseases including synucleinopathies. Moreover, further investigation of the mechanisms, by which T cells might modulate CNS myeloid cell activation, would open additional possibilities for new treatment strategies.

## Conclusions

In conclusion, using a progressive synucleinopathy model, we demonstrate a critical role of T lymphocytes in enhancing the number of α-synuclein aggregates by modulation of myeloid cell activation. The prevalence of the M1 phenotype and impaired phagocytic activity in the presence of T lymphocytes result in reduced clearance of α-synuclein aggregates, thereby enhancing progression of synucleinopathy. Thus, blocking M1 activation or an infiltration of T lymphocytes might slow disease progression and could be an option for potential pharmaceutical intervention.

## Abbreviations

CNS, central nervous system; DA, dopaminergic; Iba1, ionized calcium-binding adapter molecule 1; MPTP, 1-methyl-4-phenyl-1,2,3,6-tetrahydropyridine; PD, Parkinson’s disease; Rag2, recombination activating gene 2; SN, substantia nigra; tg, transgenic; TH, tyrosine hydroxylase; WTS, wild-type α-synuclein

## Additional files

Additional file 1: B lymphocytes do not infiltrate the midbrain of WTS^+^ Rag2^+/+^ mice. (A) WTS^+^ Rag2^+/+^ and WTS^+^ Rag2^−/−^ brains were stained for the B lymphocyte marker CD19. Representative bright field pictures of the midbrain regions show that no positive staining could be detected. (B) To proof the functionality of the applied antibody, spleen of WTS^+^ Rag2^+/+^ mice were stained with the same anti-CD 19 antibody and strong positive staining could be detected. Scale bar 50 μm. (TIF 992 kb) Additional file 2: No morphological differences were detected in CNS myeloid cells of WTS^+^ Rag2^+/+^ and WTS^+^ Rag2^−/−^ mice. (A) Representative pictures of four distinct CNS myeloid cells morphological types analyzed associated with the respective activation state of the CNS myeloid cells: type 1 CNS myeloid cells were defined as resting CNS myeloid cells, type 2 as primed, type 3 as reactive and type 4 as activated. (B) Quantification of the defined CNS myeloid cells types in the SN of WTS^+^ Rag2^+/+^ and WTS^+^ Rag2^−/−^. Scale bar 20μm. (TIF 1856 kb)
