# Supplementary figures and images for: Infiltrating T lymphocytes reduce myeloid phagocytosis activity in synucleinopathy model
Source: J Neuroinflammation. 2016 Jun 30;13:174. doi: 10.1186/s12974-016-0632-5 (PMC4929755; doi:10.1186/s12974-016-0632-5)

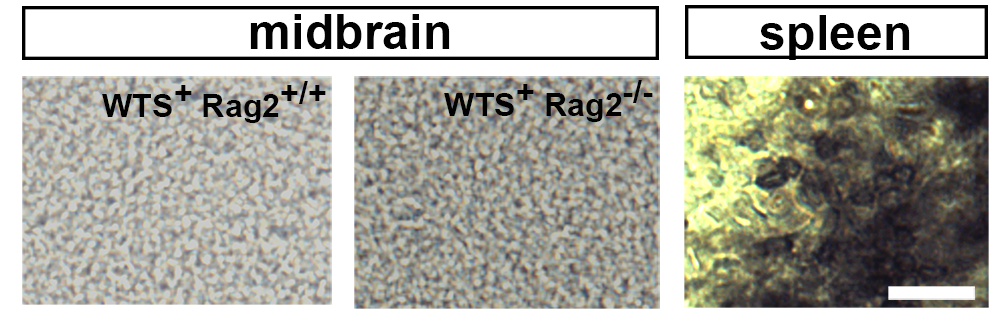

Supplement: Additional file 1: — B lymphocytes do not infiltrate the midbrain of WTS+ Rag2+/+ mice. (A) WTS+ Rag2+/+ and WTS+ Rag2−/− brains were stained for the B lymphocyte marker CD19. Representative bright field pictures of the midbrain regions show that no positive staining could be detected. (B) To proof the functionality of the applied antibody, spleen of WTS+ Rag2+/+ mice were stained with the same anti-CD 19 antibody and strong positive staining could be detected. Scale bar 50 μm. (TIF 992 kb) [file 12974_2016_632_MOESM1_ESM.tif]

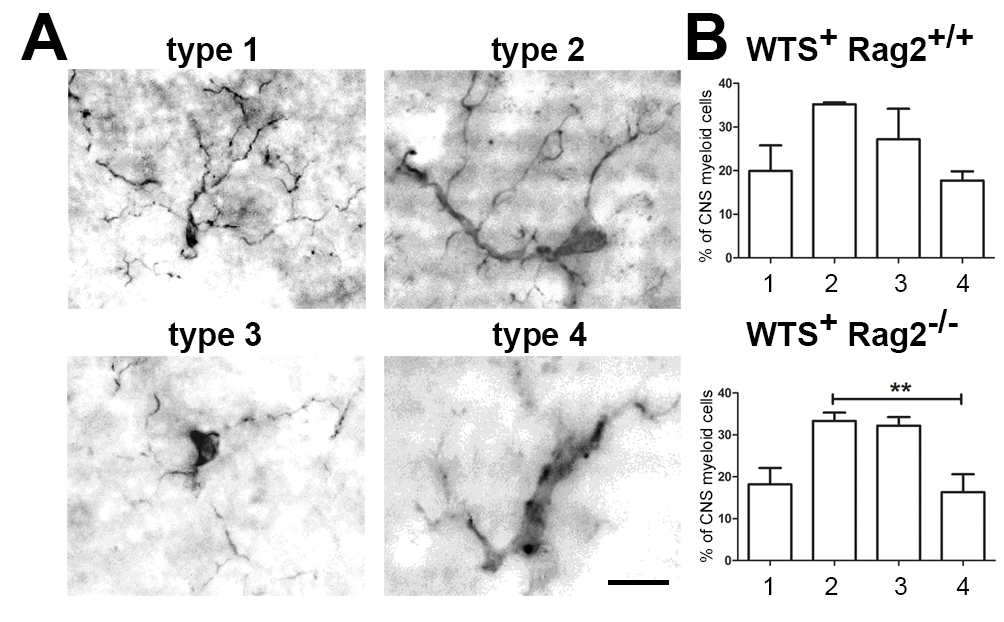

Supplement: Additional file 2: — No morphological differences were detected in CNS myeloid cells of WTS+ Rag2+/+ and WTS+ Rag2−/− mice. (A) Representative pictures of four distinct CNS myeloid cells morphological types analyzed associated with the respective activation state of the CNS myeloid cells: type 1 CNS myeloid cells were defined as resting CNS myeloid cells, type 2 as primed, type 3 as reactive and type 4 as activated. (B) Quantification of the defined CNS myeloid cells types in the SN of WTS+ Rag2+/+ and WTS+ Rag2−/−. Scale bar 20μm. (TIF 1856 kb) [file 12974_2016_632_MOESM2_ESM.tif]
